# Interview-Based Qualitative Descriptive Study on Risk Factors of School Withdrawal among Elementary School Children

**DOI:** 10.3390/children9101579

**Published:** 2022-10-19

**Authors:** Sachiko Înoue

**Affiliations:** Department of Nursing Science, Okayama Prefectural University, Soja 719-1197, Japan; sinoue@fhw.oka-pu.ac.jp

**Keywords:** school withdrawal, school refusal, elementary school children

## Abstract

With increasing school refusal among elementary school children, we qualitatively examined the associated factors. Elementary school teachers underwent semi-structured interviews, and the data generated were analyzed using SCAT. We conducted interviews with 27 teachers (18 men, nine women) responsible for children refusing to attend school. We analyzed verbatim transcripts of the interviews and abstracted six constructs for school refusal: underdeveloped interpersonal skills; families having difficulty in supporting their children to attend school; low self-esteem; diverse views on school refusal; tenuous relationships among local community members; and an environment where games and media are easily accessible. Children refusing to attend school may be unable to relate well to other people owing to underdeveloped interpersonal skills, and they may have low self-esteem. Regarding the home environment of children who are not positive about school life, living in families having difficulty in supporting their children to attend school may make the children less likely to do so. Because of tenuous relationships with local community members, there has been a decline in the involvement of people around children other than family members in encouraging school attendance. Recently, diverse views on school refusal have been accepted; increasingly, parents and children are likely to choose to spend time outside school. An environment in which games and media are easily accessible may make it easy to relate to other people without attending school, undermining the need for school attendance.

## 1. Introduction

As of 2017, the number of children refusing to attend school in Japan exceeded 35,000, accounting for 0.54% of the total number of elementary school children, and it is increasing [1]. A child with school refusal behavior is defined as “a child who is absent from school for 30 days or more in a year due to some psychological, emotional, physical, or social factor or background that causes him or her not to attend school or to be unable to attend school even if he or she wanted to, excluding those who are absent due to illness or financial reasons” [1]. However, this definition does not consider differences in reasons and background factors: it defines school refusal as any case in which a child is absent from school for 30 days or more in a year, excluding illness and financial reasons. Thus, school refusal refers to a state of non-attendance excluding a state of illness or need of welfare among chronic absentees.

In a survey on the present situation of school refusal conducted by the Ministry of Education, Culture, Sports, Science and Technology called “Recognition of the Current Status of Non-attendance at School”, many instances of school refusal were reportedly triggered by friendships and difficulty understanding studies, which previously accounted for most cases. Sudden changes in a family’s living environment (including financial problems, father or mother moving away for work, family separation, or parents changing or losing their jobs) accounted for about 10% of cases [1]. A systematic review conducted in 2018 observed factors associated with a higher risk for chronic absenteeism [2]; however, that study applied a wider definition than school refusal (i.e., it could have included cases of absence due to illness or welfare needs). Those factors include the following: individual factors, such as bullying or perceived lack of safety at school; low academic achievement; mental health conditions (e.g., attention-deficit or hyperactivity disorder, anxiety, depression, and behavioral problems); family factors, such as health problems (e.g., maternal depression); lack of structure or supervision; parents working night shifts or having multiple jobs; and school and community factors, such as economic disadvantages and unsafe routes to school [3,4,5,6,7,8,9,10,11,12,13]. However, varied pathways result in school refusal. For example, smartphone addiction has been related to academic performance failure and connected to chronic school absence [14]. Another study reported a relationship between school nonattendance and autism spectrum disorder; it identified a range of underlying reasons, including truancy, school exclusion, family decisions not to attend school, and non-problematic absenteeism [15]. However, little research has provided an interpretation of the relationship between these factors and school refusal.

Many studies have found that such factors as parent-child relationships, financial problems at home, and parents’ physical or mental illness may affect school attendance. The situation in Japan is reportedly similar [16,17]. There, aspects of the family situation are often influenced by changes in the family’s social environment and socioeconomic conditions; they are considered to change with time rather than existing universally. Thus, the current background needs to be considered when developing measures to prevent school refusal in Japan. The present study aimed to interview teachers involved in supporting nonattendance and to descriptively identify factors that triggered nonattendance and its background factors. From previous research, we inferred that school refusal is affected by social environment. Therefore, by determining the social environmental factors that influenced school refusal, it would be possible to clarify those factors.

## 2. Materials and Methods

### 2.1. Participants

In this study, we conducted semi-structured interviews with elementary school teachers. The participants were teachers working at public elementary schools in A Prefecture, Japan. The target elementary schools included small to large facilities across that prefecture and included diverse regional characteristics, such as urban, mountainous, and industrial areas. Many junior high school students who refuse to attend school have not attended school since elementary level; thus, teachers may not know the circumstances that led to the students’ school refusal. The proportion of new refusal cases among elementary school students has increased in recent years, so for this study we selected elementary school teachers with experience in dealing with student nonattendance. We requested the cooperation of the principals of public elementary schools in A Prefecture that had confirmed having had one or more students refusing to attend school over the previous 3 years. The principals who stated they would cooperate were asked to recommend one teacher who was involved in supporting students refusing to attend school. Only suitably experienced teachers were assigned to this role at each school. Our study subjects were teachers who dealt with children refusing to attend school on a regular basis.

### 2.2. Survey

The survey was conducted through direct semi-structured interviews by researchers after obtaining permission to use an IC recorder for recording (Table 1). The semi-structured interviews included questions about the following: teachers’ experience in dealing with children refusing to attend school; what they perceived about the students refusing to attend school when dealing with them; and what they considered the children were trying to express by refusing to attend school. The subjects were interviewed individually; the content was transcribed verbatim and analyzed descriptively using a qualitative inductive study design.

### 2.3. Analysis

We analyzed the text data transcribed verbatim using the steps for coding and theorization (SCAT) method. SCAT consists of describing segmented data using four-step coding by adding codes in the following order: (1) words to be focused on in the data; (2) words outside the data for rephrasing; (3) phrases for explanation; and (4) themes and constructs that emerge from the data, describing the storyline by combining the themes and constructs, and describing the theory (concept) from the storyline [18]. In this study, we do not use the term “construct” to identify the factors in school refusal; however, we abstracted the codes added to the themes in step (4) such as the factors in school refusal, and we presented the storyline by combining the codes (factors) of those themes.

The participant recruitment period lasted three months (October to December 2017). We repeated the data collection and verbatim record analysis in the above order from the collected data. If no new information emerged from the three interviewed participants, we assumed data saturation, and data collection stopped. Of the five members of the research team, one was responsible for the semi-structured participant interviews. That individual has continuously conducted research about supporting children refusing to attend school and has experience with such children, their families, and teachers in clinical settings. The other four members participated as analysis support personnel; they were students at a nursing school and were primarily responsible for analyzing the verbatim data. During the study period, no members of the research term had any contact with specific children refusing to attend school or their families who were supporting children refusing to attend school.

The analysis was conducted after SCAT training by the researchers. With SCAT, we used no analysis software. In the interviews, we asked participants about factors behind school nonattendance and focused on phrases consistently used to explain nonattendance in the responses. We did so by reviewing the transcripts and extracting potentially relevant words that could be reasons or triggers for school refusal. We used those words to generate codes. We further abstracted those codes and generated thematic phrases that better described the codes. The extracted codes and themes were determined by the researcher alone without confirming with the participants under the supervision of a qualitative analysis expert.

In this study, possible reflexivity was the influence on the data analysis by the first author’s experience in helping children not attending school. The analysis concentrated on the researcher being aware of the reflexivity of the analysis under the supervision of the qualitative research specialist. Notably, the researcher undertook repeated review and analysis in the coding and phrasing processes for consistency and discrepancies between data and experience using the first author’s experience in helping children with school refusal. Finally, under the supervision of the qualitative research expert (throughout the analysis), a storyline was generated by repeatedly checking the presence or absence of temporal relationships and interrelationships among the codes eventually generated.

### 2.4. Ethical Issues

The interviewees received a detailed explanation of the study and were asked in advance whether they would participate; they were informed about confidentiality and protection of personal and privacy information. We conducted this study after consultation with and approval by the Ethics Committee of Okayama Prefectural University (receipt number: 16–30, approved on 29 July 2016).

## 3. Results

The participants comprised 27 individuals (18 men, nine women). They included teachers who were involved in supporting children refusing to attend school on a regular basis (such as classroom teachers), school nurses, chiefs of school affairs, and teachers primarily in charge of school statistics related to school refusal. Of the 27 participants, only one was an assistant principal (i.e., management position); the others were not in such roles. Four were school nurses, and two were chiefs of school affairs; however, all the participants were responsible for student guidance. The interviews lasted 30–90 min. No participants underwent more than one interview; none withdrew from the study after the interview. Data saturation was reached after interviewing 24 participants. No new information emerged from the three participants who were subsequently interviewed, and we terminated the interviews after 27 participants had been interviewed. We stopped further participant recruitment when only repeated information arose from the final three participants.

After analyzing the verbatim data using SCAT, we extracted 292 words for focus; they were further abstracted into 22 codes by assigning words for rephrasing and adding explanatory phrases. Based on those 22 codes (indicated in italics below), we generated six themes (indicated in bold) about current school refusal (Table 2). Quotations from the verbatim data are presented in double quotation marks.

For **underdeveloped interpersonal skills**, “having no friends to talk to in interpersonal relationships among girls” and “difficulty in asserting oneself in a group” were explained as *maladjustment to complex interpersonal relationships*. “Because of developmental characteristics, people impulsively use violence against others, and there are situations where it is difficult to build relationships through verbal exchanges” and “difficulty in asking someone to do something that one wants them to do” was interpreted as *discord with surroundings because of limitation of verbal self-expression* and “feeling bullied” as *the existence of invasive interpersonal relationships*. The phrase *Children cannot cope with situations that do not go their way* was abstracted from “Children can spend their time as they want and meet their demands at home, but they cannot put themselves first in a group” and “For those who never attended nursery school and experienced group interpersonal relationships for the first time in elementary school, group relationships require communication and negotiation with people other than mothers and family members, and it is a struggle”.

For **families having difficulty in supporting their children to attend school**, *Parents cannot persuade their children* was abstracted from “Parents do not have the ability to tell their children what to do” and “Parents let their children do what they want”. “Parents are too tired from work to care for their children’s lives”, “Parents are unable to adjust their daily schedule to their children’s school schedule”, and “Parents do not help their children with studies or homework” were used as an explanatory phrase for *the tendency for parents to neglect their children because they are too busy with their own lives.* We abstracted a situation in which *Children’s life rhythm is maladjusted because of parents’ irregular lifestyles and busy schedule* from “Parents are not home when the child wakes up in the morning”, “Parents are away most of the time, and children have to prepare their own breakfast and dinner”, “Parents come home late at night, so they cannot discipline their children in daily life”, “Parents themselves work at night and are not home in the morning, so they cannot send their children to school”, and “Working parents and single parents cannot spend time with their children because they lead a different daily schedule from that of their children”.

We derived an isolated child-raising environment without extended family support and weak community relationships from “difficulty taking care of the child adequately because there is no one around to help”. Another phrase related to “The family’s low income made it impossible to meet basic needs (e.g., clothing, food, housing)” was explained by “With low income, children’s schools are not the top priority for families”, “There are situations where you have to worry about food, clothing, and shelter”, “The reason is often unknown at first, but some families with children who do not attend school need welfare support”, and “Single parents tend to have insufficient household income, giving priority to daily living and giving up sending their children to school”.

For **low self-esteem**, we abstracted *children’s feeling that their parents do not give them enough affection* from “Children feel a lack of affection because they are not cared for by their parents”, and “Parents cannot prioritize the time they spend with their children” emerged as an explanatory phrase. Similarly, we derived *children’s difficulty to adequately rely on their parents* from “Children cannot get enough of their parents”, “Children are reserved because they see their parents busy working”, and “Children do not know how to rely on their parents”. We abstracted *with control of children by parents* from “The child suppresses herself because she puts her parent’s feelings and thoughts ahead of her own”, “When a parent underestimates a child’s abilities and tries to put her under her parent’s control, the child loses self-awareness and affirmation”, and “Children try to be honor students to meet their parents’ expectations and be recognized by their parents”. *Children perceived others’ negative reactions to family members* was explained by “Seeing their parents’ unemployment status and situations where they cannot live a satisfying life because their income does not increase even if they work, children perceive negative reactions toward their families and parents from those around them and perceive them as negative reactions toward themselves”. We abstracted *Children lacked intrinsic motivation to go to school* from “Children understand that working hard does not lead to a satisfying life, and they find themselves in a situation where they do not see the meaning of learning or the meaning of going to school”.

For **diverse views on school refusal**, we derived *changes in the values and perceptions of compulsory education* from “Parents think that children do not need to attend school” as an explanatory phrase. Likewise, we abstracted *permissive home education policy about school refusal* from parents’ belief that “Children can attend school when they want to” and “There is no need to force them to attend school”; *Parents’ parenting methods for their children were influenced by their own upbringing* derived from “parents’ experiences of school refusal and their belief that there is no need to force their children to attend school”. We abstracted “Foreign countries have different education systems, and families of foreign nationals may not be able to adjust to compulsory education in Japan” from the explanatory phrase *reluctance to adapt to the Japanese educational system owing to differences from their country of origin*.

For **tenuous relationships among local community members**, “Neighbors used to talk to one another when children were at home during the day, but nowadays they are indifferent to one another” explained the phrase *a situation in which local residents no longer cared about one another.* We abstracted the phrase *a situation in which families no longer cared about how the family is regarded by the community* from “no longer care about how my family is viewed by the community” and “decreased interest in how others perceive the family in the community”. Similarly, “Socializing with neighbors, such as greeting children in the neighborhood on a regular basis and talking about child rearing, has decreased”, “Neighbors no longer help one another with child rearing”, “decreased interaction with the neighborhood on a regular basis”, “There is no interaction like the old days in areas where nuclear families gather”, and “People are so busy with their own families that they do not have time to interact with the community” were explained by *Social interaction among community members is weak, and the idea of raising children in the community has faded.*

For **an environment where games and media are easily accessible**, *Children feel at home in games that do not require direct human interaction* was abstracted from “They can interact online only when they feel like it” and “They can interact only with people they feel comfortable with”. Likewise, *children’s familiarity with online interpersonal relationships* was abstracted from “They can easily reject or break interpersonal relationships they do not like” and “They find online interpersonal relationships easier than in real life, whereas building real relationships is difficult and troublesome”.

## 4. Discussion

### 4.1. Key Study Results

This study aimed to descriptively identify factors that triggered nonattendance and its background factors, focusing on the current social environment. After the verbatim data using SCAT, we abstracted six themes about current school refusal: **underdeveloped interpersonal skills; families having difficulty in supporting their children to attend school; low self-esteem; diverse views on school refusal; tenuous relationships among local community members; and an environment where games and media are easily accessible**.

### 4.2. Six Identified Factors Contributing to School Refusal

For **undeveloped interpersonal skills**, *discord with surroundings because of limited verbal self-expression* was generated. Interpersonal skills are usually related to communication skills. People with characteristics of communication disorders (such as autism spectrum disorders) also have difficulty acquiring interpersonal skills and are prone to situations where they cannot fully express themselves [19]. We were unable to determine if these possible underlying causes of undeveloped interpersonal skills, such as *maladjustment to complex interpersonal relationships*, were present in children who refused to go to school; however, some findings demonstrate the reliability of the interpretation that children with autism spectrum disorder are more likely to be absentees [15].

In terms of *children’s inability to cope with situations that do not go their way*, a child’s words and actions that are accepted by parents and in limited interpersonal relationships at home may not be accepted as desirable in school groups. This may be attributed to the fact that the children are unable to communicate their opinions to others because of their underdeveloped interpersonal skills with language [20]. Psychosocial risk factors, such as contact problems with peers and family and school problems, often play an important part [21]. Therefore, underdeveloped interpersonal skills may be related to difficulty building good relationships at school as well as internal difficulties for the individual child and family environment.

In terms of **families having difficulty in supporting their children to attend school**, the explanatory phrases “Parents cannot persuade their children” and “Parents tend to neglect their children because they are too busy with their own lives” and a situation in which parents do not notice that their children do not get up in the morning and cannot get to school on time suggested parents avoiding confrontation with their children to support them attending school. Instead, the parents allowed their children to stay at home by accepting their statements that they did not want to attend school. Alternatively, parents may not interact with their children at all after they leave home for school. In such families, there may be a low capacity to care for the children—especially if the parents are in such situations as being exhausted from living and raising children as a single parent and having difficulty in engaging with their children and being unable to meet basic needs for food, clothing, and housing because of a low income. Laissez-faire child-raising methods without a framework within the family may also make it difficult for children to switch between home and school life because they do not have limits on time or place. According to Fukuda et al., in a study conducted in the 1980s, over 70% of school refusals, which often occurred during adolescence, were accompanied by irregularities in daily life rhythms [22]. An important factor identified in a recent study was that in many families, mothers often engage in part-time or other forms of employment when their children reach elementary school age; that contributes to neglect of the children (e.g., no meals prepared for children at home and absence of guardians at night) [23]. The employment environment in Japan has changed: the proportion of non-regular workers has increased [24]. Parents are obliged to prioritize work in order to live—even if they cannot earn enough income; as a result, they may neglect taking care of their children. The non-establishment of basic regular lifestyles in Japan today is influenced by problems in children’s behavioral development and by such factors as the home environment, parents’ work, and parents’ lifestyles. One study reported that good family functioning was associated with not refusing to attend school [25]. In the present study, we were unable to determine if families having difficulty in supporting their children to attend school functioned well or not; however, the presence of the family affects children’s schooling behavior.

For **low self-esteem**, first, we found that school refusal may be due to children sensing their own unimportance and underestimating their worth. Children feel worthless if they believe they are unloved by their parents [26,27]. Not being able to recognize one’s worth is thought to lead to a decline in self-esteem. However, other research on low self-esteem has shown that doting, overly controlling, overly interfering parents force their children to over-adapt [28]. Over-adaptation refers to being excessively adaptive through balanced adaptation; it has been reported that some children try to exceed the expectations of those around them and become overly self-controlled [29]. One report revealed similar results: children’s anxiety-related diagnoses were more strongly associated with negatively reinforced school refusal behavior; children who had separation anxiety disorder displayed attention-seeking behavior [30]. It has also been observed that anxious parents are keen to educate and discipline their children, resulting in over-interference [31]. In addition, modern women may try to be “perfect mothers” because they care about their children and try their best to be fully involved with them [32]. However, approaching a relationship with excessive affection, leading to domination, may distract children from thinking, reduce their spontaneity, and lead to low self-esteem.

Conversely, our participants reported such factors as the children’s own perception of others’ negative reactions to family members and lack of internal motivation to attend school having been caused by difficulty in finding meaning in school attendance. This may be because children were unable to relate to the idea that attending school could lead to a positive future based on their own family situation or their parents’ inability to live a financially and mentally satisfying life despite their attending school. Such children may understand the reactions to their families of the people around them and perceive them as negative reactions toward themselves, thereby resulting in school refusal. This finding is similar to one that revealed a school refusal behavior group had the most maladaptive profile and showed lower mean scores for self-concept [33]. As noted above, changes in the social and working environment may alter a family’s socioeconomic conditions, which may affect children’s development and thoughts and hinder them developing a sense of self-esteem, which is a characteristic background factor in school refusal. Some studies have also shown that high self-esteem correlates with better academic performance [34]; it decreases the development of behavioral and emotional problems, enabling more positive interpersonal relationships to be established [35]. The *lack of internal motivation to attend school* may explain children’s low self-esteem, which could decrease those positive outcomes and lead to school refusal.

We heard **diverse views on school refusal**. We abstracted the values and perceptions factors associated with compulsory education changes and with permissiveness in home education policy towards school refusal. A study reported that counselors and teachers in Japan believed that children should not be forced to do anything [36]; the same applied to parents, who may feel uncomfortable making children attend school if they do not want to do so. It has also been observed that some parents believe school is like a business, where they pay a tuition fee for their children’s education, and teachers are like store clerks [36].

Parents’ parenting methods for their children were influenced by their own upbringing and by their reluctance to adapt to the Japanese educational system owing to differences with their country of origin. Some parents may consider school education optional and cannot accept the nature of compulsory education. That could explain the increasing number of free schools that respect children’s freedom and autonomy [37]. In addition, parents and children in families who migrated to Japan from abroad may not find it meaningful to receive education or attend school because of differences in cultural backgrounds and educational systems with their home countries. Such families may also lack understanding about compulsory education in Japan. The number of incorporated free schools has increased since the 2000s [38]. Further, the number of children of foreign nationality receiving education in Japan has risen owing to foreign technical intern trainees (institutionalized in 1993) having families in Japan. A study that examined teachers’ perspectives on immigration-related barriers to family-school collaboration found that teachers noted many families lacked the resources necessary for school engagement and were hesitant to deal with schools because of required screening procedures [39]. Despite the diverse views on Japan’s educational system, it is necessary for teachers to be aware of underlying barriers to children and families regarding school attendance.

In terms of **tenuous relationships among local community members**, the following phrase was generated: *Social interaction among community members is weak, and the idea of raising children in the community has faded*. Research on community social capital suggests it is positively correlated with children’s pro-social behavior [40]. That study concluded that greater community-level social capital benefits children through increased pro-social behavior. Parents’ chance to seek help from other parents of children attending the same school is one aspect of parental social capital. One study found that parental social capital was more highly associated with children’s present belongingness to their school, society, and country [41]. That research evaluated only the effect of school social capital among parents; it did not include the wider community outside the school. However, the study found that weak social capital may affect children’s nonattendance at school.

Another phrase was generated: *Social interaction among community members is weak, and the idea of raising children in the community has faded*. One report found that residents in newly developed residential areas had weaker ties and less community attachment [42]: changes in community relationships were thought to have reduced awareness of raising children in the community. A survey was conducted by Japan’s Ministry of Education, Culture, Sports, Science and Technology on the education capacity of local communities; it observed that individualism was becoming more prevalent among parents, and the tendency not to appreciate the involvement of other people was a factor in the declining education capacity of local communities [43]. These tenuous relationships among local community members may have influenced the waning of a community’s child-rearing capacity and reduced the role of the community in supporting children’s school attendance. Thus, weak social capital or decreased interpersonal relationships in the community may reduce support for families and, consequently, may also diminish support for children’s schooling.

In terms of **an environment where games and media are easily accessible**, *child feels at home in games that do not require direct human interaction* appeared to explain the advantages and disadvantages of online interpersonal relationships. Games in which people engage with others online have increased; thus, socializing becomes easier than with direct interpersonal interactions, and that may inhibit the development of real-life relationships with other individuals. One study reported the adverse effect of social media use [44]. Although online interaction is possible at any time, it is also easy to stop or refuse such interaction. By contrast, real-life interpersonal relationships in school are complex; becoming accustomed to indirect relationships online may make it more difficult to adapt to real-life interpersonal relationships.

One study, however, observed that with online interaction, there is no need to show one’s body or face when speaking, which places less emphasis on the body; the use of interactive technology enhances self-esteem, promotes good relationships, and provides a tool for meeting and giving support to other people [45]. Thus, children not attending school may be encouraged to stay at home and keep contact with others online. Accordingly, *children’s familiarity with online interpersonal relationships* may push students to play games and maintain online interpersonal relationships at home instead of going to school to see classmates.

### 4.3. Storyline

Children refusing to attend school may be unable to relate well to others owing to **underdeveloped interpersonal skills** and may have **low self-esteem**. Regarding the home environment of children who are not positive about school life, living with **families having difficulty in supporting their children to attend school** may be less likely to lead to school attendance. Owing to **tenuous relationships with local community members**, there has been a decrease in the involvement of people around children other than family members in encouraging attendance at school. Today, **diverse views on school refusal** are accepted; increasingly, parents and children are likely to choose spending time outside school. Moreover, **an environment where games and media are easily accessible** may make it easy to relate to others without attending school, making it difficult to feel the need to attend school.

### 4.4. Study Limitations

First, our interviews were conducted with teachers, and factors related to school refusal were revealed and discussed from the results obtained. The teachers’ comments were transcribed verbatim and analyzed, and it is possible that the thoughts and actual situations of the children and their families differed from the report contents. Our results should be interpreted in this light.

Second, not all the relevant factors need be present: one factor could trigger school refusal. We were unable to examine the relationships among the factors and whether or not they were sequential. With respect to the storyline, it may be that each factor is continuous with and influences the others; however, some factors may work in a complementary manner in causing children to refuse school attendance. Further research is needed in this regard.

Third, it is necessary to consider the possibility that the interviewed teachers’ experiences in supporting children refusing to attend school may have been limited to perceivable problems. We selected suitably experienced teachers for this study, but we did not collect demographic information about the participants other than sex.

Fourth, the aim of this study was to clarify the current state of school refusal in part of Japan. We investigated school refusal among elementary school students before COVID-19, and so we did not consider social changes or new health issues related to the pandemic.

## 5. Conclusions

This study was conducted to qualitatively analyze the factors in school refusal among elementary school children. We were able to abstract six factors: underdeveloped interpersonal skills; families having difficulty in supporting their children to attend school; low self-esteem; tenuous relationships among local community members; diverse views on school refusal; and an environment where games and media are easily accessible.

## Figures and Tables

**Table 1:** Contents of the semi-structured interview guide.

Experiences of dealing with children who do not attend school.
2. Concerns and worries about dealing with children who do not attend school.
3. Your perceptions of the child when you spoke to the child about school refusal.
4. Feelings from the child’s surrounding environment and parents when responding.
5. From the experiences you dealt with, what do you feel that children’s refusal to attend school represents?

**Table 2:** Six identified factors contributing to school refusal.

Theme (Code Obtained in Step 4)	Phrases Describing Notable Phrases from the Verbatim Transcripts (Code Obtained in Step 3)
Underdeveloped interpersonal skills	Maladjustment to complex interpersonal relationships Discord with surroundings because of limitation of verbal self-expression Children cannot cope with situations that do not go their way
Families who have difficulty providing school attendance support	4. Parents cannot persuade their children 5. Children’s life rhythm is maladjusted because of parents’ irregular lifestyles and busy schedules 6. Laissez-faire child-raising methods without a framework within the family 7. Parents tend to neglect their children because they are too busy with their own lives 8. An isolated child-raising environment without extended family support and weak community relationships 9. Parents exhausted from living as a single parent and raising children, and therefore cannot be fully involved with their children 10. Children perceived disharmony in the family, giving rise to a continuing state of excessive stress 11. The family’s low income made it impossible to meet basic needs (e.g., clothing, food, housing)
Low self-esteem	12. Children could not worth themselves and underestimate of their worth 13. Parents controlled their children 14. Children lacked intrinsic motivation to go to school
Diverse views on school refusal	15. Values and perceptions associated with compulsory education were changing 16. Permissiveness home education policy about school refusal 17. Parents’ parenting methods for their children were influenced by their own upbringing 18. Reluctance to adapt to the Japanese educational system due to differences from their country of origin
The tenuous relationships among local community members	19. Local residents no longer cared about each other, and families no longer cared about appearances what the family is looked like by the community 20. Social interaction among community members was weak, and the idea of raising children in the community had faded
An environment where games and media are easily accessible	21. Children feel at home in games that do not require direct human interaction 22. Children’s familiarity with online-interpersonal relationships
